# Genome-wide Meta-analysis on the Sense of Smell Among US Older Adults

**DOI:** 10.1097/MD.0000000000001892

**Published:** 2015-10-30

**Authors:** Jing Dong, Jingyun Yang, Greg Tranah, Nora Franceschini, Neeta Parimi, Gorka Alkorta-Aranburu, Zongli Xu, Alvaro Alonso, Steven R. Cummings, Myriam Fornage, Xuemei Huang, Stephen Kritchevsky, Yongmei Liu, Stephanie London, Liang Niu, Robert S. Wilson, Philip L. De Jager, Lei Yu, Andrew B. Singleton, Tamara Harris, Thomas H. Mosley, Jayant M. Pinto, David A. Bennett, Honglei Chen

**Affiliations:** From the Epidemiology Branch (JD, ZX, SL, HC) and Biostatistics Branch (LN), National Institute of Environmental Health Sciences, Research Triangle Park, NC; Rush Alzheimer's Disease Center, Rush University Medical Center, Chicago, IL (JY, RSW, LY, DAB); California Pacific Medical Center Research Institute, San Francisco, CA (GT, NP, SC); Department of Epidemiology, University of North Carolina Gillings School of Global Public Health, Chapel Hill, NC (NF); Department of Human Genetics, University of Chicago, Chicago, IL (GA-A); Division of Epidemiology and Community Health, School of Public Health, University of Minnesota, Minneapolis, MN (AA); Institute of Molecular Medicine and Human Genetics Center, University of Texas Health Science Center at Houston, Houston, TX (MF); Pennsylvania State University-Milton S. Hershey Medical Center, Hershey, PA (XH); Sticht Center on Aging (SK) and Division of Public Health Sciences (YL), Wake Forest School of Medicine, Winston-Salem, NC; Program in Translational Neuro Psychiatric Genomics, Departments of Neurology and Psychiatry, Institute for the Neurosciences, Brigham and Women's Hospital; Harvard Medical School; Program in Medical and Population Genetics, Broad Institute, Boston, MA (PLD); Laboratory of Neurogenetics (ABS) and Laboratory of Epidemiology, Demography, and Biometry (TH), National Institute on Aging, Bethesda, MD; Division of Geriatrics, Department of Medicine, University of Mississippi Medical Center, Jackson, MS (THM); Section of Otolaryngology—Head and Neck Surgery, Department of Surgery, The University of Chicago Medicine and Biological Sciences, Chicago, IL (JMP); Department of Neurological Sciences, Rush University Medical Center, Chicago, IL, USA (JY, LY, DAB); and Departments of Neurological Sciences and Behavioral Sciences, Rush University Medical Center, Chicago, IL, USA (RSW).

## Abstract

Olfactory dysfunction is common among older adults and affects their safety, nutrition, quality of life, and mortality. More importantly, the decreased sense of smell is an early symptom of neurodegenerative diseases such as Parkinson disease (PD) and Alzheimer disease. However, the genetic determinants for the sense of smell have been poorly investigated. We here performed the first genome-wide meta-analysis on the sense of smell among 6252 US older adults of European descent from the Atherosclerosis Risk in Communities (ARIC) study, the Health, Aging, and Body Composition (Health ABC) study, and the Religious Orders Study and the Rush Memory and Aging Project (ROS/MAP). Genome-wide association study analysis was performed first by individual cohorts and then meta-analyzed using fixed-effect models with inverse variance weights. Although no SNPs reached genome-wide statistical significance, we identified 13 loci with suggestive evidence for an association with the sense of smell (*P*_meta_ < 1 × 10^−5^). Of these, 2 SNPs at chromosome 17q21.31 (rs199443 in *NSF*, *P* = 3.02 × 10^−6^; and rs2732614 in *KIAA1267*–*LRRC37A*, *P* = 6.65 × 10^−6^) exhibited *cis* effects on the expression of microtubule-associated protein tau (*MAPT*, 17q21.31) in 447 frontal-cortex samples obtained postmortem and profiled by RNA-seq (*P* < 1 × 10^−15^). Gene-based and pathway-enrichment analyses further implicated *MAPT* in regulating the sense of smell in older adults. Similar results were obtained after excluding participants who reported a physician-diagnosed PD or use of PD medications. In conclusion, we provide preliminary evidence that the *MAPT* locus may play a role in regulating the sense of smell in older adults and therefore offer a potential genetic link between poor sense of smell and major neurodegenerative diseases.

## Abstract 2

Supplemental Digital Content is available in the text

## INTRODUCTION

The human olfactory system provides us a remarkable ability to detect and discriminate odorants, and warns us of spoiled foods or dangerous environments. Olfactory dysfunction may not only result in poorer nutritional status and adverse effects on safety and quality of life, but is associated with higher mortality.^1,2^

The sense of smell decreases with age. Poor sense of smell affects over 20% of US older adults.^3^ Previous research suggests that the loss of sense of smell is among the earliest symptoms of neurodegenerative diseases such as Parkinson disease (PD)^4,5^ and Alzheimer disease (AD).^6^ For PD, this observation is consistent with the Braak hypothesis that Lewy body deposition first affects the olfactory bulb and lower brain stem before spreading into substantia nigra and cortex.^7^ Moreover, as the nasal cavity directly interacts with the environment, a dual-hit hypothesis was proposed that environmental neuro-toxicants may initiate PD development in the olfactory structures decades before PD clinical diagnosis.^8^ Therefore, research on the sense of smell in older adults may lead to a better understanding of the natural history and etiology of PD and potentially for other neurodegenerative diseases.

However, the genetic basis for the loss of the sense of smell is largely unknown. Limited studies have suggested that genetic variations at human olfactory receptors or the *ApoE* ε4 allele may contribute to variable olfactory sensitivity and perception.^9,10^ Two earlier studies also suggest potential links to regions on chromosome 4q.^11,12^

Here, we report the first ever genome-wide meta-analysis on the sense of smell among 6252 US older adults of European descent from 3 cohorts: the Atherosclerosis Risk in Communities (ARIC) study, the Health, Aging, and Body Composition (Health ABC) study, and the Religious Orders Study and the Rush Memory and Aging Project (ROS/MAP). We further examined suggestive loci with gene-based analyses, pathway-enrichment analyses, as well as expression quantitative trait loci (eQTL) analysis using 447 postmortem prefrontal-cortex tissues. Overall, our results suggest that microtubule-associated protein tau (*MAPT*), a major susceptibility gene for PD^13,14^ and possibly for AD,^15^ may also play a role in regulating the sense of smell in older adults.

## METHODS

### Participating Studies

Details of participating cohorts were published elsewhere.^16–19^ Briefly, the ARIC study is an ongoing longitudinal study that was established in 1987 to 1989 to investigate risk factors for cardiovascular diseases.^16^ The sense of smell was measured as part of the ARIC Neuro-Cognitive Study (NCS) in 2011 to 2013. Of the 6523 participants of ARIC NCS, 4066 were eligible after excluding non-Whites (n = 1563), and participants without valid data on the smell identification test (n = 279) or genome-wide association study (GWAS) genotyping (n = 615). The Health ABC is a prospective study established in 1997 to 1998 to investigate risk factors for disability and functional decline among older adults.^17^ The sense of smell was tested among 2537 participants in 1999 to 2000. After excluding self-reported non-Whites (n = 976), participants without valid data on the smell identification test (n = 106) or GWAS (n = 111), 1344 were eligible for analysis. The ROS and MAP studies are longitudinal cohorts established in mid-1990s to investigate aging and AD incidence and progression among adults 65 years or older.^18,19^ The sense of smell was measured as part of its clinical evaluation. Our analysis included 1065 Whites after excluding participants without data on genotyping (n = 661) or the sense of smell (n = 644).

### The Smell Identification Tests

All 3 cohorts used validated smell identification tests to examine the sense of smell: the 12-item Sniffin’ Sticks screening test (Burghart, Wedel, Germany)^20^ in ARIC and the 12-item Brief Smell Identification Test (B-SIT, Sensonics, Haddon Heights, NJ)^21^ in the Health ABC and ROS/MAP. Both tests assess participant's sense of smell to correctly identify 12 daily odorants, although the exact odorants were somewhat different. Briefly, participants were instructed to smell each odorant and choose the correct odorant from 4 possible answers in a multiple-choice format. One point was given for each correct answer with a total score from 0 to 12. Both tests have been validated and widely used in clinical and epidemiological studies.^20,21^

All cohorts assessed global cognitive function during the same study visit in which the sense of smell was evaluated. The ARIC and ROS/MAP studies used the Mini-Mental State Examination (MMSE) that has a maximal score of 30, whereas the Health ABC study used the Modified Mini-Mental Status Examination (MMMSE) with a maximal score of 100. Each cohort also determined *ApoE* genotypes by genotyping 2 *ApoE* variants at codons 130 and 176 (formerly 112 and 158) separately.

### Genotyping, Imputation, and Statistical Analysis

GWAS genotyping was performed using the Affymetrix Genome-wide Human SNP array 6.0 in ARIC and ROS/MAP, and Illumina Human1M-Duo BeadChip in Health ABC. Imputation was performed in each cohort using MACH based on HapMap Phase II CEU build 36 reference panel. Prespecified criteria were used for quality control of each cohort.

Each cohort performed its own GWAS analysis following a standardized procedure with ProbABEL^22^ or PLINK,^23^ using a linear regression model with analysis of allele dosages. The outcome was the natural log of the sense of smell score plus 1 to account for the few participants who had a score of 0. Potential confounders were adjusted for in 2 separate models: first for age, gender, study sites, cognitive function, and the first 2 principal components (PCAs) in Model 1, and then additionally for *ApoE* ε4 allele in Model 2. We further adjusted for *ApoE* ε4 because it is the most important genetic risk factor for AD and predicts poor sense of smell in all participating cohorts.

We performed meta-analysis using a fixed-effect model with inverse variance weights in METAL,^24^ after filtering out SNPs with MAF < 1% ^25–27^ or low imputation quality (r^2^ < 0.3). We applied genomic control for each dataset before meta-analysis (e-Methods). We considered *P* value < 5 × 10^−8^ as genome-wide significant and *P* value between 1 × 10^−5^ and 5 × 10^−8^ as suggestive evidence for an association.

We used VEGAS^28^ (Versatile Gene-based Association Study) for gene-based analysis and calculated individual gene-wise *P* values for each gene in relation to the sense of smell, and used binomial test to identity enriched ingenuity canonical pathways (http://www.ingenuity.com; IPA, Ingenuity^®^ Systems) based on the results of meta-analysis in Model 2, the enrichment test *P* values were derived from 10,000 rounds of permutation tests (e-Methods).

To explore the functional relevance of top SNPs from the GWAS analysis, we applied the publicly available data from the RegulomeDB (http://regulomedb.org/)^29^ (e-Methods). We then generated RNA expression data using frozen postmortem dorsolateral prefrontal cortex (DLPFC) brain tissue from 447 ROS/MAP participants to define the biological consequence of the risk variants of identified SNPs (e-Methods).

### Ethical Aspects

Individual study protocols were approved by relevant institutional review boards and all study participants provided written consent.

## RESULTS

Table 1 presents characteristics of study participants. As expected, the sense of smell decreased with age and was lower in men than women. Further, higher cognitive score was associated with better sense of smell, whereas the *ApoE* ε4 allele was related to poorer sense of smell. These population characteristics were similarly associated with the sense of smell across cohorts (Table S1, http://links.lww.com/MD/A526). Although these studies used different screening tests of smell identification, the distributions of the sense of smell scores were comparable across cohorts (Figure S1, http://links.lww.com/MD/A526).

**TABLE 1:**
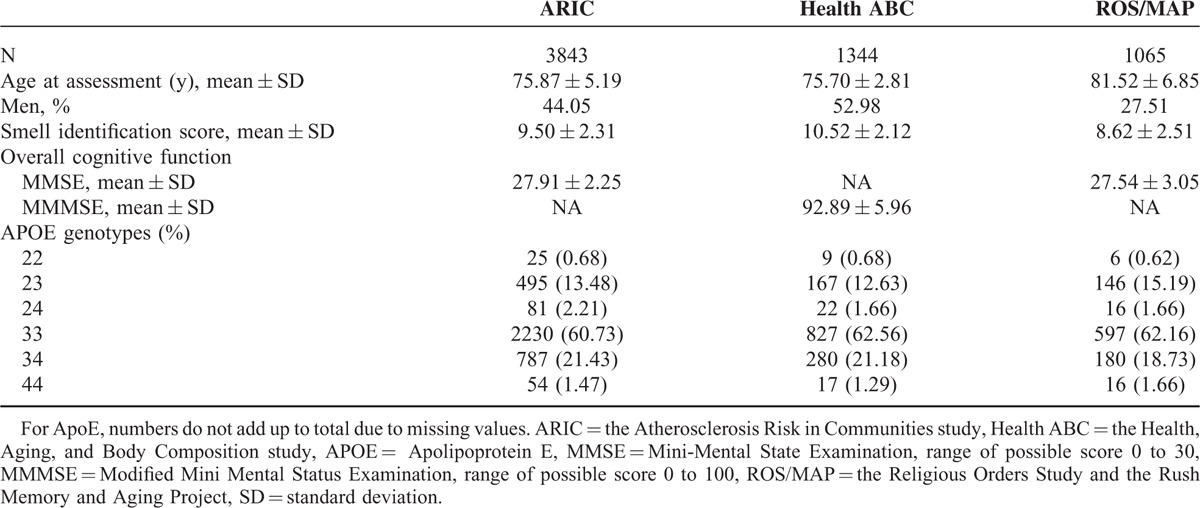
Population Characteristics of Participating Cohorts

**FIGURE 1:**
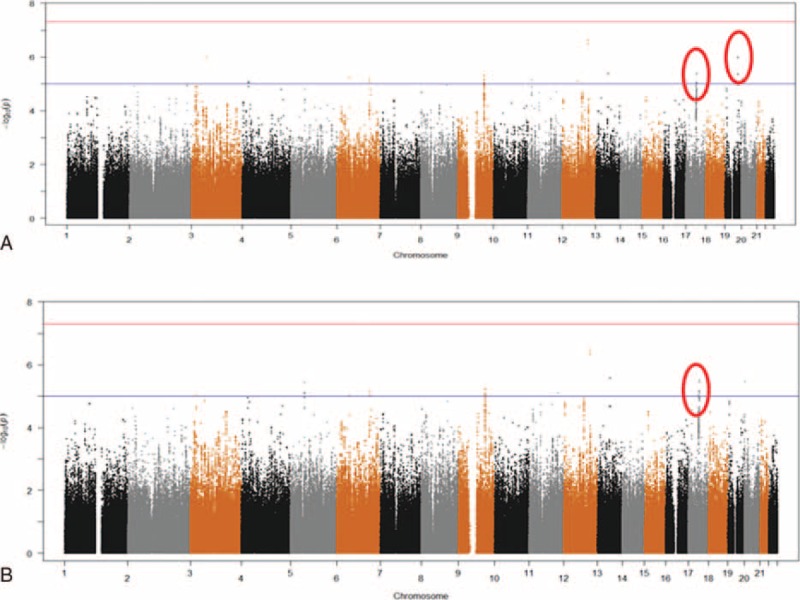
Manhattan plots of the genome-wide meta-analysis on the sense of smell. The red line indicates the genome-wide significance threshold (5 × 10^−8^) and the blue line indicates the suggestive threshold (1 × 10^−5^). (A) Model 1 adjusted for age, gender, study center, cognitive function, and the first 2 principal components; (B) Model 2 further adjusted for *ApoE* ε4 allele.

The Q–Q plots for Model 1 showed relatively low genomic inflation factors (overall λ = 0.996), suggesting minimal population stratification (Figure S2, http://links.lww.com/MD/A526). Although no SNP reached genome-wide significance, 35 SNPs had a suggestive association with the sense of smell (*P*_meta_ < 1 × 10^−5^, Figure 1A). We examined LD among these SNPs and identified 19 SNPs that either had the lowest *P* value in each LD block (r^2^ ≥ 0.8) or were potentially functional as annotated in the RegulombDB (Tables S2 and S3, http://links.lww.com/MD/A526). Among these, 3 SNPs were predicted as eQTLs in frontal-cortex and cerebellum tissues in the RegulomeDB: rs199443 (*NSF*, *P*_meta_ = 4.01 × 10^−6^), rs2075650 (*TOMM40*, *P*_meta_ = 4.24 × 10^−6^), and rs2732614 (*KIAA1267*–*LRRC37A*, *P*_meta_ = 8.34 × 10^−6^). The rs199443 and rs2732614 were *cis*-eQTLs for *MAPT* in both frontal-cortex and cerebellum tissues,^30^ while rs2075650 was *cis*-eQTL for *TOMM40* in lymphoblastoid cells.^31^

In the meta-analysis of Model 2 that further adjusted for *ApoE* ε4, the Q–Q plot also showed a relatively low genomic inflation factor with an overall λ of 0.996 (Figure S2, http://links.lww.com/MD/A526). As shown in Table 2 and Figure 1B, 13 SNPs had suggestive associations with the sense of smell (*P*_meta_ < 1 × 10^−5^, Figure S3, http://links.lww.com/MD/A526); another 47 SNPs were in LD (r^2^ ≥ 0.8) with these 13 SNPs (Table S4, http://links.lww.com/MD/A526). Ten of these 13 SNPs overlapped with those from Model 1, including the 2 annotated eQTLs at chromosome 17: rs199443 (*P*_meta_ = 3.02 × 10^−6^) and rs2732614 (*P*_meta_ = 6.65 × 10^−6^). In contrast, the signal for rs2075650 at *TOMM40* was drastically diminished (*P*_meta_ = 0.09) after adjustment for *ApoE* ε4. For these 13 SNPs, we also repeated the meta-analyses using random-effect model and observed similar results. For example, the *P*_meta_ was 3.73 × 10^−6^ for rs199443 and 8.25 × 10^−6^ for rs2732614 under the random-effect model.

**TABLE 2:**
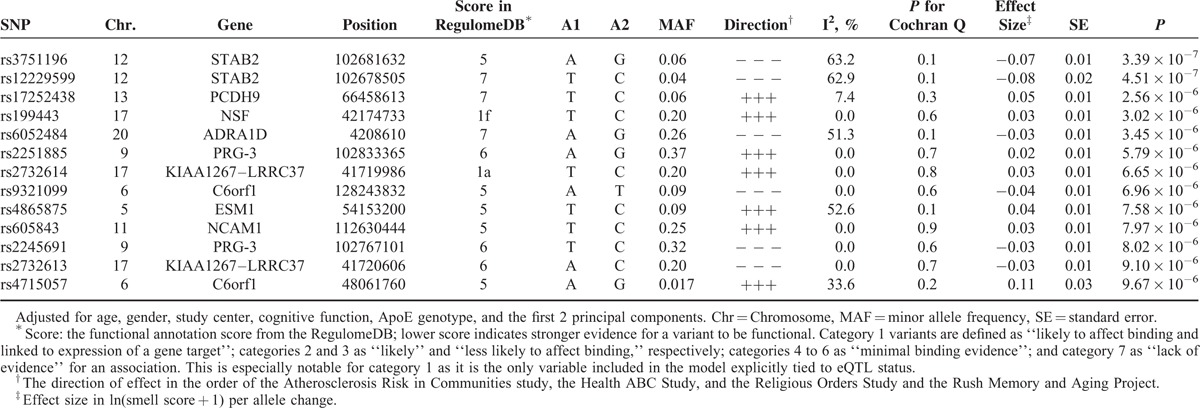
Top Ranked SNPs (*P*_meta_ < 1 × 10^−5^) From the Genome-Wide Meta-Analysis on the Sense of Smell in Model 2

We conducted gene-based analysis using VEGAS^28^ to identify genes that harbor multiple association signals based on *P*_meta_ values from the model that adjusted for *ApoE*. The SNPs were mapped to 17,693 genes. No genes were statistically significant after Bonferroni correction (*P* < 2.8 × 10^−6^ = 0.05/17,693). Genes with *P* < 1.0 × 10^−3^ were listed in Table S5, http://links.lww.com/MD/A526. Interestingly, *MAPT* (*P*_gene_ = 9.4 × 10^−5^) was among the top genes identified in this analysis along with nearby *LRRC37A* (*P*_gene_ = 2.9 × 10^−5^) and *NSF* (*P*_gene_ = 1.4 × 10^−5^), all located on chromosome 17. *LRRC37A* and *NSF* each harbor 1 of the 2 annotated eQTLs (rs2732614 and rs199443, respectively) whereas the expression of *MAPT* is regulated by both SNPs in brain tissues.^30^

We further conducted 2 pathway enrichment analyses (top 10 identified pathways listed in Table S6, http://links.lww.com/MD/A526). The first analysis was based on results from Model 2 and we observed 21 significantly enriched canonical pathways with the permutation *P* < 0.05. These include the CDK5 and p38 MAPK signaling pathways both of which are implicated in neurodegeneration and *MAPT* is a member of both pathways. The second analysis were based on the 164 genes with *P*_gene_ < 0.01 identified by VEGAS. This analysis identified 113 enriched canonical pathways with the permutation *P* < 0.05. The CDK5 signaling pathway was again identified as 1 of the top ranked pathways.

**FIGURE 2:**
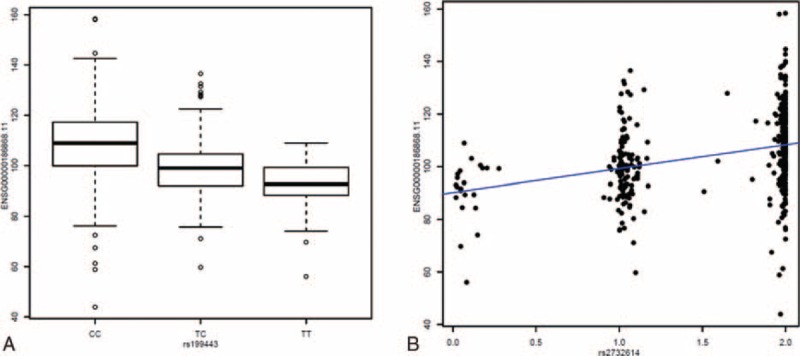
Expression quantitative trait loci analysis for MAPT in 447 human frontal cortex samples. These plots illustrate a dose relationship between allele load at rs199443 (A) and rs2732614 (B) and expression of *MAPT*; the *P* values were 2.22 × 10^−16^ and 4.44 × 10^−16^, respectively. For rs2732614, genotype data were imputed using MACH which provides an estimated dosage of imputed genotype. The dosage ranges from 0 to 2, with 0 as no copy of the reference allele (C allele) and 2 as 2 copies of the reference allele. MAPT = microtubule-associated protein tau.

We further examined *MAPT* expression using RNA-seq data of 447 postmortem frontal-cortex tissues from the ROS/MAP study in order to define the biological consequence of the risk variants of rs2732614 and rs199443. We found strong correlations between genotypes of the risk allele (C allele for both rs2732614 and rs199443) and higher expression level of *MAPT* (*P* < 10^−15^, Figure 2); however, we observed no significant correlation between these 2 SNPs and the expression levels of *NSF* or *LRRC37A* (rs2732614: *P* = 0.62 for *NSF* and 0.54 for *LRRC37A*; rs199443: *P* = 0.79 for *NSF* and 0.11 for *LRRC37A*).

As *MAPT* is 1 of the most important susceptibility genes for PD, we further performed a sensitivity analysis for the 13 SNPs identified in Model 2 by removing potential PD cases based on self-reported physician diagnosis or use of PD medications (90 in ARIC, 14 in Health ABC, and 12 in ROS/MAP) and obtained similar results (Table S7, http://links.lww.com/MD/A526).

## DISCUSSION

To the best of our knowledge, this is the first genome-wide meta-analysis on the sense of smell among older adults. The study population includes 3 well-characterized aging cohorts with objective measurements for smell identification. Although no SNP reached genome-wide significance, the preponderance of the evidence suggests *MAPT* as a potential susceptibility locus for poor sense of smell. This is particular interesting because *MAPT* is 1 of the most important susceptibility genes for late-onset sporadic PD^13,14^ and possibly for AD.^15^

Smell impairment is common among older adults, particularly in patients with neurodegenerative diseases. More importantly, recent data suggest that decreased sense of smell may precede the clinical onset of both PD^4,5^ and AD.^6^ For example, in the Honolulu Asia Aging Study, individuals in the lowest quartile of the sense of smell score were 5 times more likely to develop PD in the next 4 years than those in the highest quartile^5^; further poor sense of smell was associated with higher prevalence of incidental Lewy bodies in postmortem brains, a condition that may represent prodromal PD.^32^ Similar observations have also been made for both mild cognitive impairment and AD.^6,33^ Therefore, the declining sense of smell in older adults may indicate underlying neurodegenerative processes. Characterizing the genetic and environmental determinants for the sense of smell in the context of aging may provide targets for intervention with enormous potential translational impact.

The human *MAPT* locus has long been associated with the risk for PD.^13,14^ A recent meta-analysis of GWAS data further implicated *MAPT* as a susceptibility locus for late onset sporadic AD.^15^ The human *MAPT* encodes the microtubule-associated protein tau. It is notable that tau-related pathology was found in the bulbar component of the anterior olfactory nucleus only in patients of neurodegenerative disorders with marked loss of the sense of smell, such as AD, PD, and Lewy body dementia.^34,35^ Experimental studies further suggest that overexpression of tau may contribute to olfactory dysfunction in vivo.^36,37^ These findings indicate that overexpression or abnormal aggregation of tau may represent a common mechanism between olfactory dysfunction and several neurodegenerative diseases.

The genomic architecture in the region spanning *MAPT* is highly complex. It covers ∼1.8 Mb block of LD encompassing not only *MAPT* but also several other genes that have been linked to neurodegenerative diseases, such as *STH* (Saitohin) and *NSF* (N-ethylmaleimide sensitive factor). We, however, in the RNA-seq data of 447 postmortem frontal-cortex tissues, found that rs2732614 and rs199443 had statistically significant *cis* effects on the expression of *MAPT* (*P* < 10^−15^), but not on *NSF* or *LRRC37A*. Additional gene-based and pathway-enrichment analyses also showed the potential of *MAPT* as a candidate susceptibility gene for poor sense of smell. Although our study provides fairly consistent evidence that *MAPT* may be a candidate susceptibility locus for the sense of smell in older adults of European descent, fine-scale mapping of this LD block with next generation sequencing will be necessary to identify potential causal locus.

*ApoE* ε4 is the most important genetic risk factor for late-onset sporadic AD^38^ and has been linked to olfactory dysfunction in several studies.^9^ The present study confirms that the *ApoE* ε4 allele is a risk factor for poor smell identification even after accounting for cognitive function. In our initial analysis, we also identified an intronic SNP in *TOMM40* (rs2075650) in association with the sense of smell; but this signal was extinguished after adjustment for the *ApoE* ε4 allele. Although the mechanisms are yet unknown, preliminary studies showed that ApoE was expressed at high levels in various types of cells in the olfactory epithelium and its underlying lamina propria, and that *ApoE* might play a critical role in olfactory nerve regeneration in mice.^39,40^ Furthermore, experimental studies suggest that in contrast to the *ApoE* ε2 and ε3 alleles, the *ApoE* ε4 fails to promote neurite outgrowth in olfactory epithelium cultures, which may lead to olfactory dysfunction in AD.^41^

The main limitation of the current study was the lack of independent confirmation of GWAS signals. GWAS analysis often requires very large sample size to obtain adequate power to detect modest effects of underlying variants in complex diseases. However, few large population-based studies of older adults have both the sense of smell and GWAS data. We were able to identify data from 3 well-established cohorts with similar population characteristics and data collection. We chose to combine our samples for SNP discovery rather than set up the conventional 2-stage design to maximize power to detect signals. We did, however, follow up suggestive signals with functional and expression analyses. Finally, the determinants for the sense of smell are likely complex^42,43^ and our study was conducted among old White adults, therefore the results should be interpreted in this context. Further studies are needed to confirm our findings and to examine whether they could be generalized to other populations.

In conclusion, we report the results of the first comprehensive GWAS analysis to understand the genetic basis for the sense of smell in older adults of European ancestry. We provide novel evidence that the *MAPT* locus may regulate the sense of smell in older adults. This finding warrants independent confirmation and further mechanistic investigations into how *MAPT* region contributes to the loss of the sense of smell and the development of related neurodegenerative diseases.
